# Effective bioremediation of clarithromycin and diclofenac in wastewater by microbes and *Arundo* donax L

**DOI:** 10.1007/s11356-023-27660-4

**Published:** 2023-05-30

**Authors:** Laura Ercoli, Rudy Rossetto, Sabrina Di Giorgi, Andrea Raffaelli, Marco Nuti, Elisa Pellegrino

**Affiliations:** 1 Crop Science Research Center (CSRC), Scuola Superiore Sant’Anna, Piazza Martiri Della Liberta 33, 56127 Pisa, Italy; 2 Ministero Della Salute, Direzione Generale per l’Igiene e la Sicurezza degli Alimenti e della Nutrizione, Rome, Italy

**Keywords:** 4’-Hydroxydiclofenac, Bioremediation, Clarithromycin, Diclofenac, Giant reed, *Phanerochaete chrysosporium*, *Streptomyces rochei*, *Trametes versicolor*

## Abstract

Bioremediation of pharmaceuticals has gained large research efforts, but there is still a need to improve the performance of bioremediation systems by selecting effective organisms. In this study, we characterized the capability to remove clarithromycin (CLA) and diclofenac (DCF) by the bacterium *Streptomyces rochei*, and the fungi *Phanerochaete chrysosporium* and *Trametes versicolor*. The macrolide antibiotic CLA and the non-steroid anti-inflammatory DCF were selected because these are two of the most frequently detected drugs in water bodies. Growth and content of the PhCs and a DCF metabolite (MET) by the energy crop *Arundo donax* L. were also evaluated under hydroponic conditions. The removal rate (RR) by *S. rochei* increased from 24 to 40% at 10 and 100 µg CLA L^−1^, respectively, averaged over incubation times. At 144 h, the RR by *P. chrysosporium* was 84%, while by *T. versicolor* was 70 and 45% at 10 and 100 CLA µg L^−1^. The RR by *S. rochei* did not exceed 30% at 1 mg DCF L^−1^ and reached 60% at 10 mg DCF L^−1^, whereas approached 95% and 63% by *P. chrysosporium* and *T. versicolor*, respectively, at both doses. Root biomass and length of *A. donax* were strongly affected at 100 µg CLA L^−1^. CLA concentration in roots and shoots increased with the increase of the dose and translocation factor (TF) was about 1. DCF severely affected both shoot fresh weight and root length at the highest dose and concentration in roots and shoots increased with the increase of the dose. DCF concentrations were 16–19 times higher in roots than in shoots, and TF was about 0.1. MET was detected only in roots and its proportion over the parent compound decreased with the increase of the DCF dose. This study highlights the potential contribution of *A. dona*x and the tested microbial inoculants for improving the effectiveness of bioremediation systems for CLA and DCF removal.

**Supplementary Information:**

The online version contains supplementary material available at 10.1007/s11356-023-27660-4.

## Introduction

Over recent decades, the presence of pharmaceuticals compounds (PhCs) and their metabolites in the aquatic environment has been documented worldwide (e.g., Fatta-Kassinos et al. 2011; Wilkinson et al. 2022) at concentrations ranging from few ng L^−1^ to hundreds μg L^−1^ (Castiglioni et al. 2006; Gros et al. 2010; Hughes et al. 2012; Petrie et al. 2015; Wilkinson et al. 2022). Post-consumption excretion of PhCs through urine and feces by humans and animals represents the main and widespread source of PhCs released into the environment, while pharmaceutical industries are the secondary point source (Jjemba 2006; Wilkinson et al. 2018). The final concentrations of PhCs and/or their metabolites in surface water is mainly affected by the removal of the sewage treatment plants, breakdown in surface water, and dilution by river flows and rainfall (Castiglioni et al. 2006; Verlicchi et al. 2012). Although PhCs and their metabolites are often present in low concentrations in water bodies, bioaccumulation and biomagnification processes lead to an increase in detectable biologically active molecules, with toxic effects for both fauna and flora (Christensen 1998). In addition, the presence of antibiotics at low concentration in the wastewater leads to the improvement of bacteria resistance against the existing antibiotics (Baquero et al. 2008).

The removal or transformation of PhCs and their metabolites by conventional wastewater treatment plants is only partially achieved, as they were designed with the principal aim of removing easily or moderately biodegradable compounds (Verlicchi et al. 2012; Garcia-Rodríguez et al. 2014). Additional technologies (i.e., ozonation, reverse osmosis, advanced oxidation processes) can be included in the depuration process, but their high cost limits the widespread application (Göbel et al. 2005; Castiglioni et al. 2006; Grandclément et al. 2017). Therefore, it is imperative to develop nonconventional technologies with low operation and maintenance cost that are effective in PhC decontamination.

One promising technique is bioremediation, using natural biological activity (i.e., plants and microorganisms). This technology has been applied to remove/transform toxic compounds located in soils, sediments, groundwater, and surface water with varying degrees of success according to pollutant and environmental conditions (Juwarkar et al. 2010; Adams et al. 2015). Among bioremediation techniques, constructed wetlands (CWs) have been accepted as an attractive and economic alternative for the improvement of the overall effluent quality prior to discharge into surface waters (Verlicchi et al. 2013; Carvalho et al. 2014; Carvalho 2020). In these nature-based systems, macrophytes, invertebrates, and microorganisms can uptake, metabolize, or sequester PhCs and nutrients (Li et al. 2014; Ilyas and van Hullebusch 2019). However, despite the effective degradation of PhCs by CWs, one of their main limitations is the low efficiency of removal since they typically require low hydraulic loading rates, and therefore large surface areas. In addition, they need relatively long retention times, as the length of time the water is in contact with the substrate, biofilm, and plant roots affects the extent to which the removal or biotransformation of the PhCs can occur (Carvalho 2020).

Therefore, there is a strong need to improve the design of these systems for their better performance by a proper selection of the most efficient organisms, plants, and microorganisms, which tolerate the potential toxic effects of the wastewaters, and grow and uptake/degrade the toxic contaminants. An additional criterion for the choice of plants is the delivery of an economic income which can originate from the utilization of their biomass as raw materials for energy production or green chemistry (Pandey et al. 2016). The most widely used plants for remediation purposes are common reed (*Phragmites australis*) (Carvalho et al. 2012; Hijosa-Valsero et al. 2010), *Typha* spp. (Dordio et al. 2010), and giant reed (*Arundo donax*) (Elhawat et al. 2014; Coppa et al. 2020; Zhang et al. 2021). However, to our knowledge, *A. donax*, selected for this study, was not previously tested for the removal or biotransformation of PhCs. This plant species can be grown as energy crops and for phytoremediation purposes and has shown interesting yields of lignocellulosic biomass from shoots under low fertility conditions, along with a preferential allocation of contaminants in rhizomes that can be harvested at the end of the phytoremediation program (Fagnano et al. 2020; Zhang et al. 2021).

Among the PhCs most frequently detected in the aquatic environment (e.g., Gavrilescu et al. 2014; Verlicchi et al. 2012), clarithromycin (CLA), an antibiotic, and diclofenac (DCF), a non-steroidal anti-inflammatory drug, were selected as the target molecules in the present study. CLA is a macrolide antibiotic and one of the most prescribed drugs in human medicine to treat upper and lower respiratory tract infections, as well as skin and mycobacterial infections (Kummerer and Henninger 2003; Pereira et al. 2013; Cardini et al. 2021). The presence of CLA in wastewater effluents has been reported in various studies at concentrations ranging from 12 to 536 ng L^−1^ worldwide (Miao et al. 2004; Miege et al. 2009; Fatta-Kassinos et al. 2011; Verlicchi et al. 2012; Michael et al. 2013). Li et al. (2014), in a review summarizing the state of research activities on the application of CWs for removing PhCs from wastewater, showed that the removal efficiency of CLA was from 18% in conventional waste-water treatment plants (WWTPs) to 31% in CWs. Similarly, Verlicchi and Zambello (2014) estimated removal efficiency of CLA ranging from 10 to 60% in CWs, acting as secondary and tertiary steps. DCL is widely used in human and veterinary medicine to reduce inflammation and pain (Caracciolo et al. 2015). It is one of the most commonly detected PhCs in effluents of WWTPs, at concentrations ranging from < 1 to 4110 ng L^−1^ (Miege et al. 2009; Gavrilescu et al. 2014; Luo et al. 2014). The removal efficiency of DCF from conventional WWTPs widely varies from 17% (Heberer 2002) to 75% (Daughton and Ternes 1999), and higher efficiencies are associated to the addition of advanced procedures, such as chemical degradation assisted by specialized microorganisms, or UV light action. Zhang et al. (2014) in a review, summarizing the PhCs and personal care products removal performance in different aquatic plant-based systems, estimated a removal efficiency of DCF up to 87% in hybrid CW systems applied as alternative secondary treatments, and up to 98% in hybrid CWs applied as tertiary treatments.

*Trametes versicolor* and *Phanerochaete chrysosporium* are white-rot fungi able to produce extracellular enzymes (i.e., lignin peroxidases and manganese peroxidases) that can degrade lignin and are also able to mediate oxidation of a wide variety of organic pollutants and heavy metals, as a result of the non-specificity of their enzyme system (Baldrian 2008; Cameron et al. 2000). For PhCs, some experiments have demonstrated the degrading ability of white-rot fungi. Marco-Urrea et al. (2009) investigated the degradation ability of ibuprofen, clofibric acid and carbamazepine by four white-rot fungi (*T. versicolor*, *Irpex lacteus*, *Ganoderma lucidum*, and *P. chrysosporium*). Whereas ibuprofen was extensively degraded by all the fungi tested, *T. versicolor* was the only species able to degrade either cloficric acid and carbamazepine, although the latter was also degraded by *G. lucidum*. Marco-Urrea et al. (2010), investigating the degradation of DCF by *T. versicolor*, observed in liquid media almost a complete removal (≥ 94%) in about 30’ at a concentration of 10 mg L^−1^ and 45 μg L^−1^. *Trametes versicolor* was reported to be able also to degrade CLA (≥ 96%) at an initial concentration of 76 ng L^−1^ in inoculated sterile and not-sterile membrane biological reactor sludge (Llorens-Blanch et al. 2015).

The aim of this study was to characterize the microbial and plant bioremediation of CLA and DCF from aquatic media. This study presents results from two experiments. The first experiment was carried out to assess the capability of the bacteria *Streptomyces rochei*, and two fungi, *P. chrysosporium* and *T. versicolor*, to remove CLA and DCF. The second experiment aimed to evaluate plant growth and uptake of CLA and DFC by *A. donax* L. grown in mesocosm under hydroponic solution. The microbial strains used in the first experiment were *S. rochei* DSM 41,732, *P. chrysosporium* DSM 1556 and *T. versicolor* DSM 11,309, which have been considered good candidates for bioremediation because they use a wide range of C sources and are naturally occurring microorganisms in the rhizosphere of plants (Bumpus et al. 1985; Pointing 2001). The second experiment with *A. donax* L. was performed in mesocosm under hydroponic conditions to avoid the potential interference of soil or other wetland substrate particles that could adsorb the tested PhCs (Liu et al. 2013). Moreover, considering the potential utilization of the aerial plant part for energy purposes, the partitioning of CLA and DCF among plant parts has been assessed. The achieved results would provide solutions for the improvement of the design of effective and cost-efficient nature-based depuration plants to remove PhCs from wastewaters.

## Materials and methods

### Microbial degradation of CLA and DCF

#### Microbial strain propagation

**Fig. 1:**
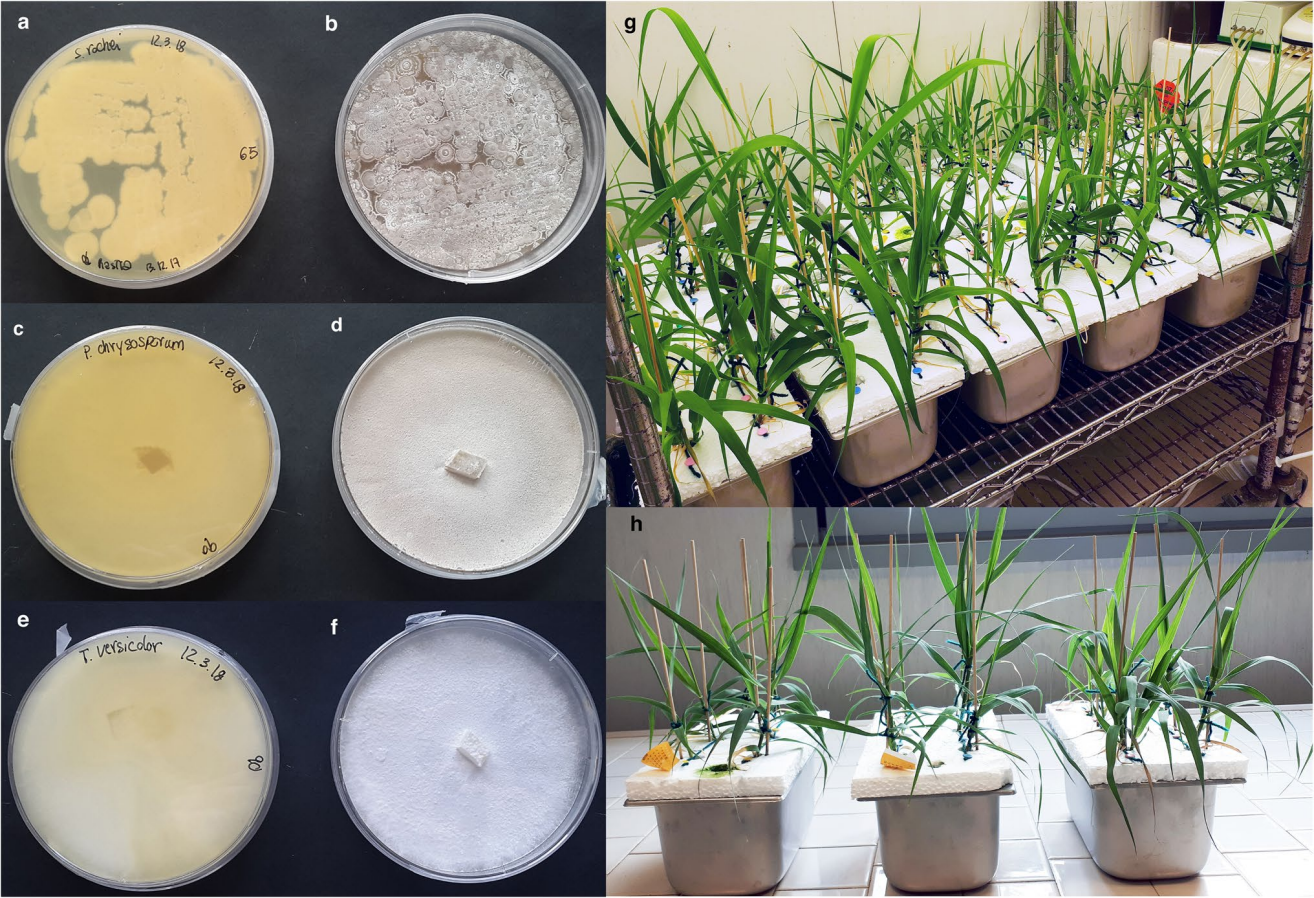
*Streptomyces rochei* DSMZ 41,732 (**a**, **b**) stock bacterium culture maintained in a GYM Streptomyces medium; *Phanaerochete chrysosporium* DSMZ 1556 (**c**, **d**) and *Trametes versicolor* DSMZ 11,309 (**e**, **f**) and stock fungal cultures maintained in malt extract peptone agar. Examples of the experimental set-up utilized for studying the *Arundo donax* growth and uptake of clarithromycin (CLA) and diclofenac (DCF) at different pharmaceutical doses and time of exposure (**g**, **h**)

*Streptomyces rochei* DSMZ 41,732 stock cultures were maintained at 28 °C in a GYM Streptomyces medium (DSMZ, medium 65), whereas *P. chrysosporium* DSMZ 1556 and *T. versicolor* DSMZ 11,309 and stock cultures were maintained at 25 °C and 35 °C, respectively, in malt extract peptone agar (medium 90) (DSMZ) (Fig. 1) (DSMZ n.d.).

#### Set-up of the experiment of CLA and DCF microbial degradation

A batch experiment was set up for assessing the efficacy of degradation of CLA and DCF by *S. rochei*, *P. chrysosporium*, and *T. versicolor*. For *S. rochei*, a spore suspension was prepared from the stock plates. The culture surface of each plate was gently scraped with a sterilized spatula, and spores were suspended in 9 mL of sterile water (Kieser et al. 2000). The suspension was filtered through a syringe with a non-absorbent cotton wool sterile filter (Termo Fisher Scientific, USA), and then, 9 mL of glycerol solution (40% v/v) were added to each tube containing the spore filtrate. The tubes were shaked in a vortex mixer to homogenize the solution and stored at − 20 °C until further use. For each suspension, additional plates on agar medium were set up in order to determine the colony forming units (c.f.u.) per mL of suspension by the method of serial dilutions. An average of 1 × 10^9^ c.f.u. of *S. rochei* per mL of solution was detected. Then, two pre-inoculum Erlenmeyer flasks (150 mL) were prepared by the addition of 1 mL of the spore suspension to 99 mL of the DSMZ medium 65. Since *S. rochei* tends to grow forming a rather compact masses or pellets in liquid medium (Hobbs et al. 1990), 50 glass beads were inserted in each flask to encourage the dispersed growth of the bacterial biomass (Kieser et al. 2000). Flasks were covered with aluminum foils and incubated at 28 °C in an orbital shaker (IKA KS 4000 I Control Shaker, Germany) at 110 rpm. The pre-inoculum of *S. rochei* was kept in the orbital shaker for 48 h. For the preparation of the fungal pre-inocula of *P. chrysosporium* and *T. versicolor*, an agar disk (ca. 5 mm in diameter) taken from the external portion of a stock culture was transferred in each flask containing 30 mL of liquid medium (DSMZ medium 90). Two flasks per each fungus were set up and incubated in the orbital shaker at 30 °C and 110 rpm for 4 days, with steel beads in order to homogenize the fungus.

The degradation experiment with the bacterial/fungal inoculum was carried out in Erlenmeyer flasks of 125 mL. The bacterial/fungal mycelium was collected from the pre-inoculum flasks, centrifuged at 3000 rpm for 10 min, washed in sterilized UHQ water, and divided in four aliquots that were then resuspended in 50 mL of the appropriate medium. Two solutions were used for the determination of the concentration of the inoculum (oven-dry weight at 50 °C), while two solutions were used as inoculum. The pre-inoculum concentration was about 2 g L^−1^ on a dry weight basis. An inoculum of ca. 10 mg of mycelium was taken from the pre-inoculation flasks and applied to the flask utilized to the degradation experiment. This corresponds to a concentration of mycelium in the inoculated flask of 0.1 g L^−1^. CLA was added to the flasks to give a final concentration of 10 e 100 µg L^−1^ (Merck, Darmstadt, Germany), while DCF (sodium salt, Cayman Chemical, Michigan, USA) was added to a final concentration of 1 mg L^−1^ and 10 mg L^−1^. To summarize, for the three microorganisms, each flask was inoculated with 5 mL of inoculum, spiked with the different doses of PhCs and filled up to 100 mL of the appropriate growth medium. Thus, the experiments of CLA/DCF microbial degradation were set-up following a full-factorial completely randomized design with two concentrations of the PhC (CLA doses: 10 and 100 µg L^−1^; DCF doses: 1 and 10 mg L^−1^) and five sampling time (*T*_inc_: 0, 24, 48, 72, and 144 h). To evaluate the possible adsorption of the PhCs by dead microorganisms, the experiment included also killed controls, which consisted of inoculated flasks that were then autoclaved at 120 °C for 20 min before the addition of the PhCs. Each treatment was replicated four times (*n* = 4); thus, a total of 48 flasks were set-up for each PhCs. All the cultures were grown at 28 °C in the orbital shaker at 110 rpm. Moreover, to exclude the possible influence of light on DCF and CLA stability, all the replicates were maintained in the dark using aluminum foil.

#### Sampling and analyses

Samples were collected from each flask at 0, 24, 48, 72, and 144 h for the determination of CLA/DCF concentration. Two mL were sampled from each flask and then centrifuged at 17,000 rpm for 20 min. Finally, the supernatant was transferred in amber HPLC vials, without moving the bacterial pellet. The LC–MS/MS analyses were performed on a PE Sciex API 365 triple quadrupole mass spectrometer (AB Sciex LLC, Framingham, MA, USA) equipped with a Turbo Ionspray source, interfaced to an Agilent 1100 HPLC system with binary pump and auto-sampler (Agilent, Santa Clara, CA, USA) (McArdell et al. 2003; Pierattini et al. 2018). The separation was carried out by a Phenomenex Synergi Fusion 2 × 75 mm column, 5 µm particle size (Phenomenex, Torrance, CA, USA) using the following chromatographic conditions: mobile phase A, acetonitrile with 0.1% formic acid, mobile phase B, water with 0.1% formic acid; gradient: flow rate, 400 µL/min; 0–1 min, A 10%; 1–8 min, A to 95%; 8–10 min, A 95%. The analytes (CLA and DCF) were determined using the SRM (selected reaction monitoring) technique, monitoring two fragmentations for each component. SRM details and retention times of the analytes are reported in the Table S1. After the LC–MS/MS analysis, the removal rate of CLA/DCF was calculated for each sampling time as [100– ([CLA/DFC]_*x*_/([CLA/DFC]_0_) × 100]. A calibration curve for DCF (Sigma-Aldrich, Germany) was built: *R*^2^ = 0.993 and concentration range 20–10,000 ng mL^−1^ DCF. Similarly, a calibration curve for CLA (Sigma-Aldrich, Germany) was built: *R*^2^ = 0.989 and concentration range 1–10,000 ng mL^−1^ CLA. Data were normalized according to matrix effect, calculated as peak area of the sample spiked after extraction/peak area of the standard, and recovery percentages of 99.7% and 98.9%, respectively, calculated as peak area of the sample spiked before extraction/peak area of the sample spiked after extraction. Limit of detection (LOD) of DCF and CLA was 1 ng mL^−1^ and 0.5 ng mL^−1^, respectively, while limit of quantification (LOQ) of DCF and CLA was 3.18 ng mL^−1^ and 1.60 ng mL^−1^.

### Plant growth and uptake of CLA and DCF by Arundo donax

#### Plant material and set up of the microcosm experiment

Plants of *A. donax* from in vitro cultures (micropropagated plants) were acclimatized for 5 weeks in a growth chamber under controlled environmental conditions (23:18 °C day:night temperature, 65/70% relative humidity, 16 h photoperiod at 400 µmol m^-2^ s^-1^ photosynthetic photon flux density supplied by fluorescent lights). During the acclimation process, 15 plants were grown in steel containers of 3 L in Hoagland nutrient solution (a total of 30 containers), continuously aerated by aquarium pumps (a total of 450 plants). Plants were held in place in the lids of the pots by a layer of polystyrene. The nutrient solution was replaced every 2 weeks.

After 5 weeks of acclimation (T0), when new roots and leaves had developed, plants of same size (approximately 10 cm height) were selected and transferred to steel containers of 3 L (8 plants per container), which contained aerated Hoagland nutrient solution spiked respectively with 10 and 100 mg L^-1^ of CLA and 1 and 10 mg L^-1^ of DCF. The control plants for each PhC were not spiked. Plants were grown in the growth chamber maintaining the controlled environmental conditions applied for acclimatation. For CLA, each treatment was replicated six times (*n* = 6), whereas for DCF, it was replicated four times (*n* = 4). The containers were arranged in a completely randomized design. An example of the experimental set-up is reported in Fig. 1g, h. To exclude the effect of root-associated microbial communities in the degradation of CLA/DCF, the plants roots were sterilized in NaClO solution (8%) before PhC application and sterility checked by plating onto Nutrient agar plates. For CLA, 18 containers were set up (3 doses × 6 replicates), whereas for DCF, 12 containers were set up (3 doses × 4 replicates). In order to avoid photodegradation of CLA and DCF, the containers were covered with aluminum foils. Moreover, abiotic controls were set up to exclude other dissipation mechanisms like volatilization, photooxidation, or adsorption.

#### Plant physiological measurements: growth parameters and chlorophyll content

Four acclimated plants per replicate were sampled at the beginning of the experiment (T0). Then, in the CLA experiment, four plants were sampled after 18 and 30 days of growth (T18 and T30, respectively), whereas in the DCF experiment, four plants were samples after 18 days of growth (T18). At each sampling, plants were carefully washed with deionized water and separated into roots and shoots (stems and leaves) for fresh weight (FW) and dry weight (DW) determination (oven dried at 70 °C to constant weight). In addition, stem and leaf number was recorded, as well as the occurrence of any visual symptom of injury. After image capture of leaves by 16 MP Samsung SM-A520F mobile phone camera, the total leaf area was determined using the open-source image processing program ImageJ (https://imagej.net/downloads), while root length was measured using the semi-automated digital image analysis tool HyLength (Cardini et al. 2020). Chlorophyll concentration in leaves was determined by a SPAD meter (SPAD-502 chlorophyll meter, Konica Minolta, Osaka, Japan).

#### Extraction and quantification of CLA/DCF from plant tissues

At the sampling times T18 and T30 in the CLA experiment and at T18 in the DCF experiment, 0.5 g of fresh roots and shoot were collected for the analysis and stored at − 80 °C until extraction for the analysis of CLA and DCF concentration in plant tissues. The four plants from individual containers were pooled in the sample collection. Plant organs (roots and shoots) were finely grounded in liquid N_2_, transferred into tubes and then weighted. Two milliliter of methanol was added to each tube. The extracts were sonicated for 5 min and centrifuged at 17,000 × g for 10 min. After centrifugation, the supernatant was filtered through a 0.45-µm syringe cellulose acetate membrane filters (Sigma-Aldrich, Germany) and stored at − 20 °C until further analysis. Quantification of CLA/DCF was made by LC–MS/MS, as described above. In addition to the analytes CLA and DCF, 4’-hydroxydiclofenac was determined. A calibration curve for DCF (Sigma-Aldrich, Germany) was built: *R*^2^ = 0.997 and concentration range 10–10,000 ng mL^−1^ DCF. Similarly, a calibration curve for CLA (Sigma-Aldrich, Germany) was built: *R*^2^ = 0.994 and concentration range 1–10,000 ng mL^−1^ CLA. Data were normalized according to matrix effect, calculated as peak area of the sample spiked after extraction/peak area of the standard, and recovery percentages of 99.5% and 99.1%, respectively, calculated as peak area of the sample spiked before extraction/peak area of the sample spiked after extraction. LOD of DCF and CLA was 0.7 ng mL^−1^ and 0.4 ng mL^−1^, while LOQ of DCF and CLA was 2.20 ng mL^−1^ and 1.27 ng mL^−1^.

Plant uptake of CLA/DCF was calculated by multiplying CLA/DCF concentrations in the plant tissues by DW. The partitioning of CLA/DCF in roots and shoots was calculated. The ability of *A. donax* plants to accumulate the studied PhCs from the nutrient solution in roots and shoots during the experiment was estimated using the bioaccumulation factor (BAF), which was calculated as the ratio of CLA/DCF concentration in roots and shoots (in fresh weight basis) and in the nutrient solution. The translocation factor (TF) was calculated as the ratio of CLA/DCF concentration in the shoots and the concentration in roots.

#### Statistical analysis of results

Data on the removal rates of CLA and DCF collected in the experiment of PhC microbial degradation were analyzed by a two-way analysis of variance (ANOVA), using dose and time of incubation as fixed factors. For the *A. donax* experiment, a two-way ANOVA was performed using CLA dose and time of exposure as fixed factors. Similarly, for the *A. donax* experiment, a one-way ANOVA was performed using the DCF dose as fixed factor. Data were ln- and arcsine-transformed when needed to fulfil the assumptions of the ANOVA. Differences between means were assessed by a post-doc Tukey *B* test. Means and standard errors given in figures and supplementary tables are for untransformed data. All these analyses were performed in SPSS version 21.0 (SPSS Inc., Chicago, Illinois, USA).

## Results

### Microbial degradation of CLA

**Fig. 2:**
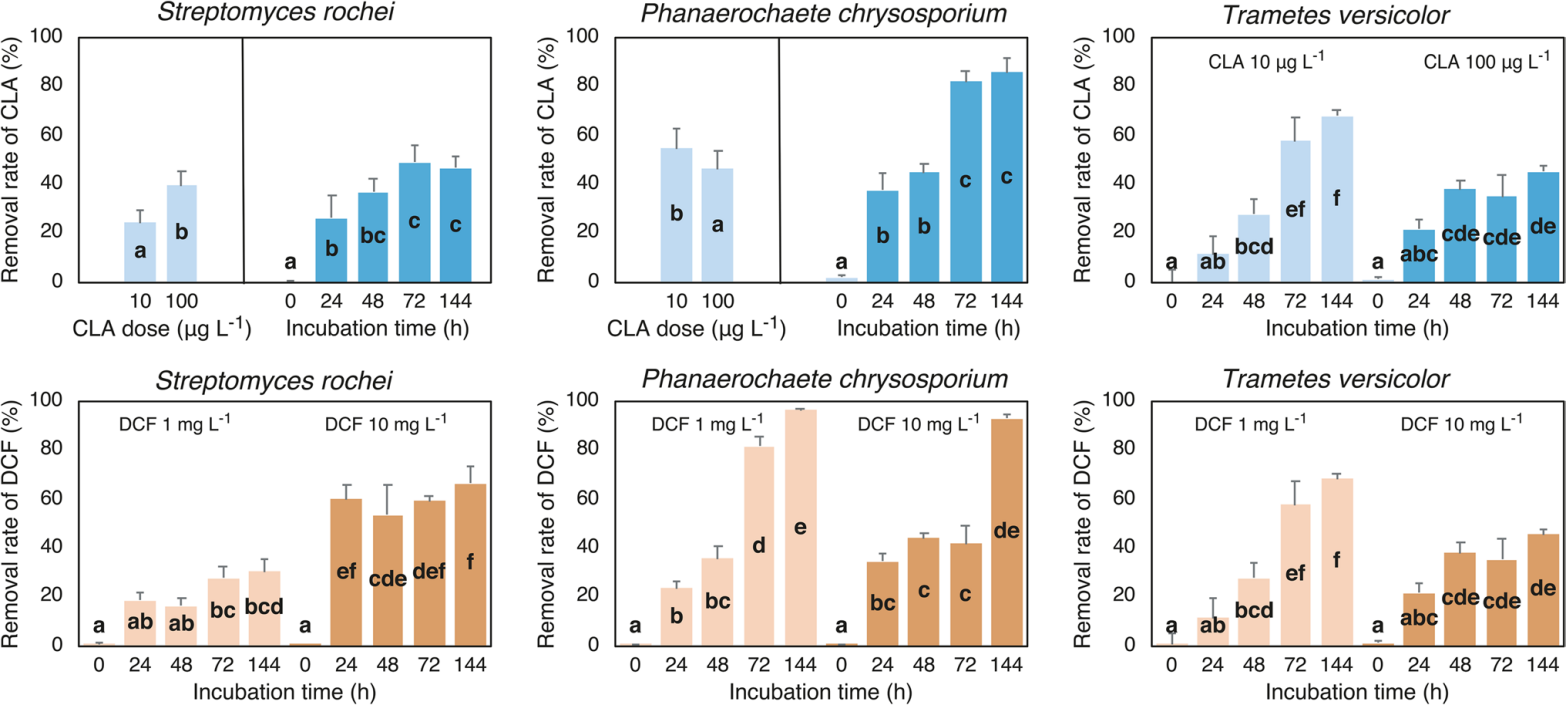
Effect of clarithromycin (CLA) concentration (dose) and time of incubation (*T*_inc_ 0, 24, 48, 72, and 144 h) on the removal rate of CLA by *Streptomyces rochei* and *Phanaerochete chrysosporium* and of the interaction between dose and *T*_inc_ on the removal rate of CLA by *Trametes versicolor*. Effect of the interaction between dose and *T*_inc_ on the removal rate of diclofenac (DCF). Concentrations in the nutrient medium were for CLA 10 and 100 µg L^−1^ and for DCF 1 and 10 mg L.^−1^. The *T*_inc_ were 0, 24, 48, 72, and 144 h. Data are mean ± SE (*n* = 4). Different letters indicate significant differences at *P* ≤ 0.05. Details about the two-way ANOVAs are given in Supplementary Table S2

In the batch experiments with *S. rochei* and *P. chrysosporium*, the removal rate of CLA was significantly affected by the main effect of PhC concentration (dose) and time of incubation (*T*_inc_), whereas with *T. versicolor*, the removal rate was affected by the interaction of the two factors (Fig. 2; Supplementary Table S2). The removal rate by *S. rochei* increased from 24% at 10 µg CLA L^−1^ to 40% at 100 µg L^−1^, and increased during incubation time until 72 h, after that it was unchanged (ca. 47%). Conversely, the removal rate by *P. chrysosporium* decreased from 55% at 10 µg CLA L^−1^ to 46% at 100 µg L^−1^, and during incubation it raised to 42% at 24 and 48 h and to 84% at 72 and 144 h. *Trametes versicolor* progressively degraded CLA with the increase of the incubation time, but the rates of increase were higher at 10 than at 100 µg CLA L^−1^. As a consequence, at 144 h, the removal rate was 70% at 10 µg CLA L^−1^ and 45% at 100 µg CLA L^−1^.

### Microbial degradation of DCF

The removal rate of DCF varied according to the interaction between dose and time of incubation (Fig. 2; and Supplementary Table S2). At all incubation times, *S. rochei* was less effective at the lower than at the higher concentration (1 and 10 mg L^−1^, respectively). At 1 mg DCF L^−1^, the removal rate was lower than 20% up to 48 h and did not exceed 30% at 72 and 144 h. At the higher concentration (10 mg DCF L^−1^), already after 24 h, the removal rate was 60%, and remained unchanged until 144 h. Conversely, *P. chrysosporium* was highly effective at both concentrations since the removal rate at 144 h approached 97% and 93% at 1 and 10 mg L^−1^, respectively. However, at the intermediate incubation time of 72 h, the removal rate was higher at 1 mg DCF L^−1^ than at 10 mg L^−1^ (82% *vs* 42%). Finally, *T. versicolor* progressively degraded DCF with the increase of incubation time up to 62–63% of the initial DCF concentration at both concentrations, but with the lowest DCF concentration, the highest removal rate was achieved already at 72 h (ca. 60%), while with the highest DCF concentration, the highest removal rate was achieved at 144 h (ca. 63%).

### Growth and uptake of CLA by Arundo donax

**Fig. 3:**
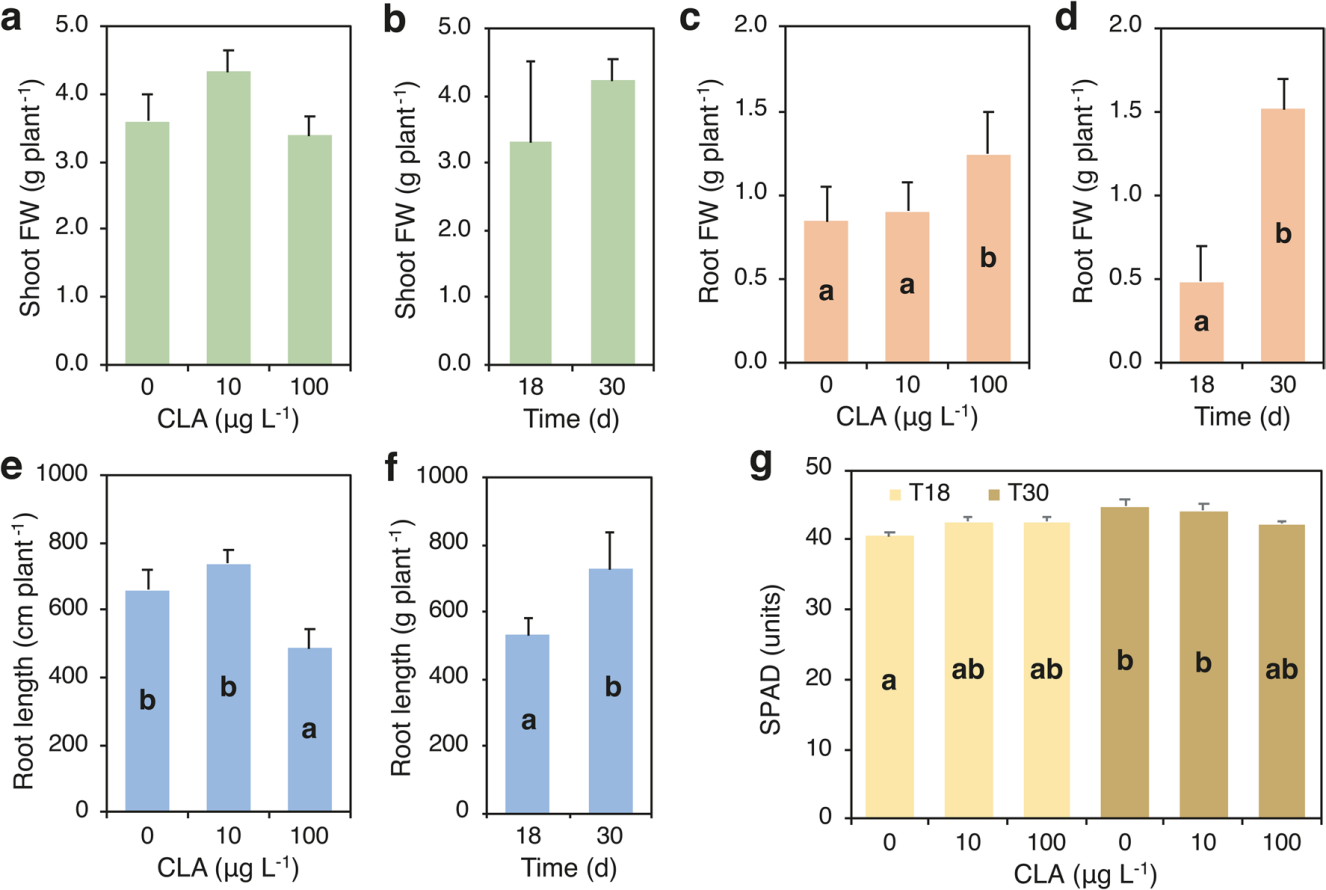
Effect of clarithromycin (CLA) concentration in the nutrient medium (0, 10, and 100 µg L.^−1^) (dose) on fresh weight of shoots (**a**) and roots (**c**), and root length (**e**) of *Arundo donax*; effect of time of exposure (18 and 30 days: T18 and T30) on fresh weight of shoots (**b**) and roots (**d**) and root length (**f**) of *Arundo donax*; effect of the interaction between CLA concentration and time of exposure on the SPAD of leaves (**g**). Data are mean ± SE (*n* = 6). Different letters indicate significant differences at *P* ≤ 0.001. Details about the two-way ANOVAs are given in Supplementary Table S3

Plants harvested just before the addition of CLA (T0) had an average of 1.2 culms per plants and 8 green leaves per plant, total fresh weight was 1.64 g plant^−1^ (shoot plus roots) and plant height was 10 cm (data not shown). Root fresh weight and root length were significantly modified by CLA concentration (dose) in the growth medium and differed over time (Fig. 3c–f; Supplementary Table S3). Averaged over time, root fresh weight at 10 µg CLA L^−1^ did not change compared to the untreated control, while it significantly increased by 48% at 100 µg CLA L^−1^ (Fig. 3c). Root length showed an opposite pattern as it was unchanged at 10 µg CLA L^−1^ respect to the untreated control and significantly decreased by 27% at 100 µg CLA L^−1^ (Fig. 3e). Thus, the morphology of the root system of *A. donax* changed in response to CLA supply: in plants treated with 10 µg CLA L^−1^ the ratio root length/root fresh weight did not change compared to the untreated plants (819.2 *vs* 787.0 cm g^−1^), whereas in plants treated with 100 µg CLA L^−1^, the ratio dropped to 391.4 cm g^−1^. Averaged over CLA dose, root fresh weight and root length increased by 215% and 37% from T18 to T30 (Fig. 3d, f). Moreover, visual observations showed that in plants treated with the highest dose of CLA, the root system consisted of only short and thick main roots.

Shoot fresh weight, leaf area, and number of stems were not affected by CLA dose and did not vary across time (Fig. 3a, b; Supplementary Table S3), whereas the number of leaves increased from T18 to T30 (13 *vs* 16 leaves) (Supplementary Table S3). Leaf SPAD readings were affected by the interaction between CLA dose and time: at T18, they slightly increased with the increase of CLA concentration in the medium, whereas at T30 values, they were higher at 0 and 10 µg CLA L^−1^ and slightly decreased (− 6%) at 100 µg CLA L^−1^ (Fig. 3g; Supplementary Table S3).

**Fig. 4:**
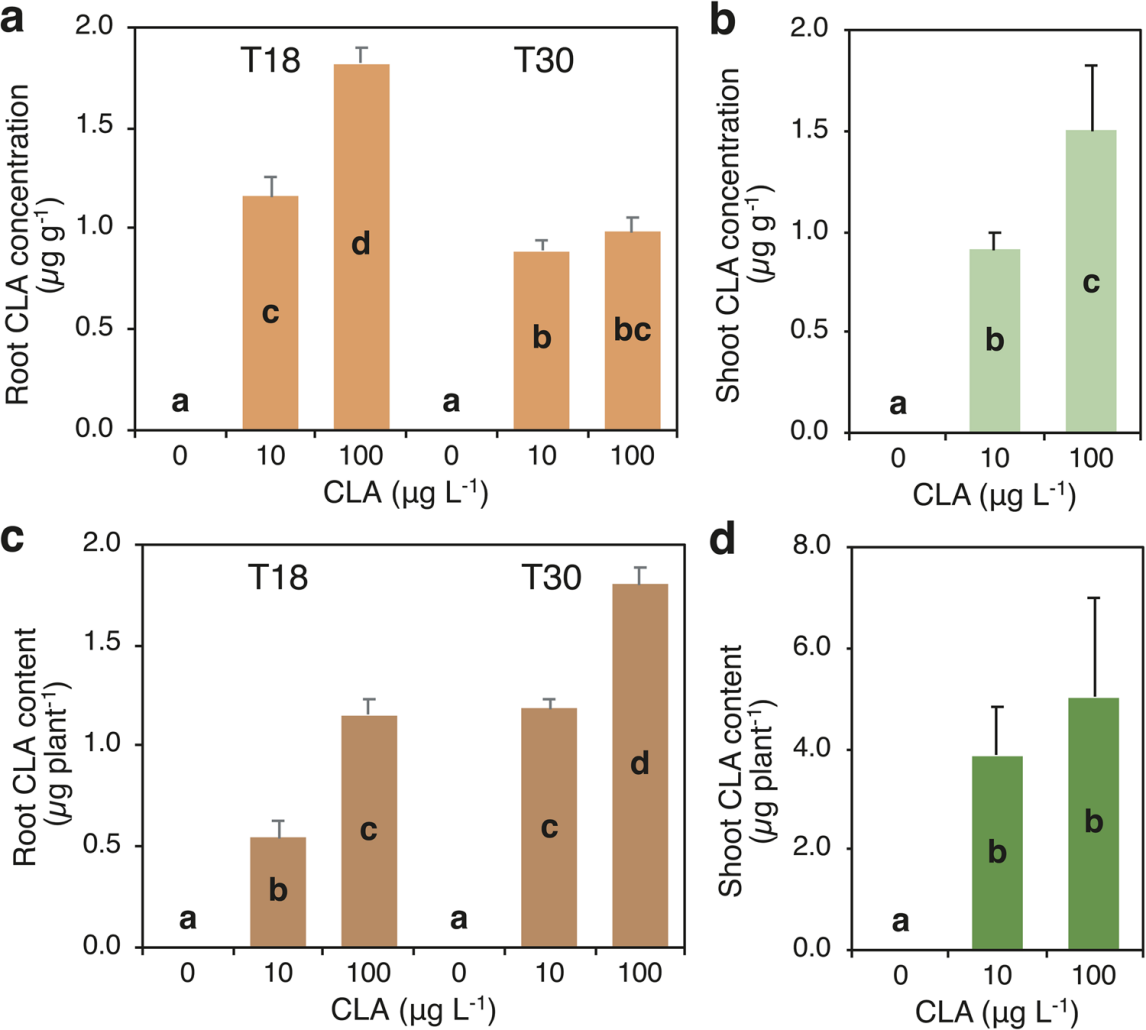
Effect of the interaction between clarithromycin (CLA) concentration in the nutrient medium (0, 10, and 100 µg L.^−1^) (dose) and time of exposure (18 and 30 days: T18 and T30) on concentration (**a**) and content (**c**) of CLA in roots; effect of CLA concentration on concentration (**b**) and content (**d**) of CLA in shoots. Data are mean ± SE (*n* = 6). Different letters indicate significant differences at *P* ≤ 0.05. Details about the two-way ANOVAs are given in Supplementary Table S4

CLA concentration and content in roots were affected by the interaction between CLA dose and time of exposure (Fig. 4a, c; Supplementary Table S4). CLA was not detected in roots of the untreated plants, while concentration of CLA in roots increased with the increase of CLA dose at T18 up to 1.8 µg g^−1^, whereas at T30 values at both doses, it did not exceed 1.8 µg g^−1^ (Fig. 4a). CLA content in roots showed a different pattern: values increased with the increase of CLA dose, but at T30, the values were significantly higher than at T18 at both CLA doses (Fig. 4c). CLA concentration and content in shoots were affected only by CLA dose and they did not show statistically significant differences between T18 and T30 (Fig. 4b, d; Supplementary Table S4). Averaged over sampling times, CLA was not detected in the shoots of the untreated plants, while shoot CLA concentration increased with the increase of the dose from 0.91 to 1.50 µg g^−1^ (Fig. 3b). Similarly, CLA content in shoots increased with CLA dose, but differences between the doses 10 and 100 µg L^−1^ were statistically not significant (on average 4.5 µg plant^−1^) (Fig. 4d). Overall, at T30, CLA content was higher in shoots than in roots at both CLA doses, about three-fold and over two-fold at 10 and 100 µg CLA L^−1^, respectively (Supplementary Table S4). CLA content at T30 in the whole plant increased from 4.6 to 6.3 µg plant^−1^ with the increase of CLA dose from 10 to 100 g L^−1^, and it was partitioned for 75% and 71% into shoots, respectively (Supplementary Table S4).

**Fig. 5:**
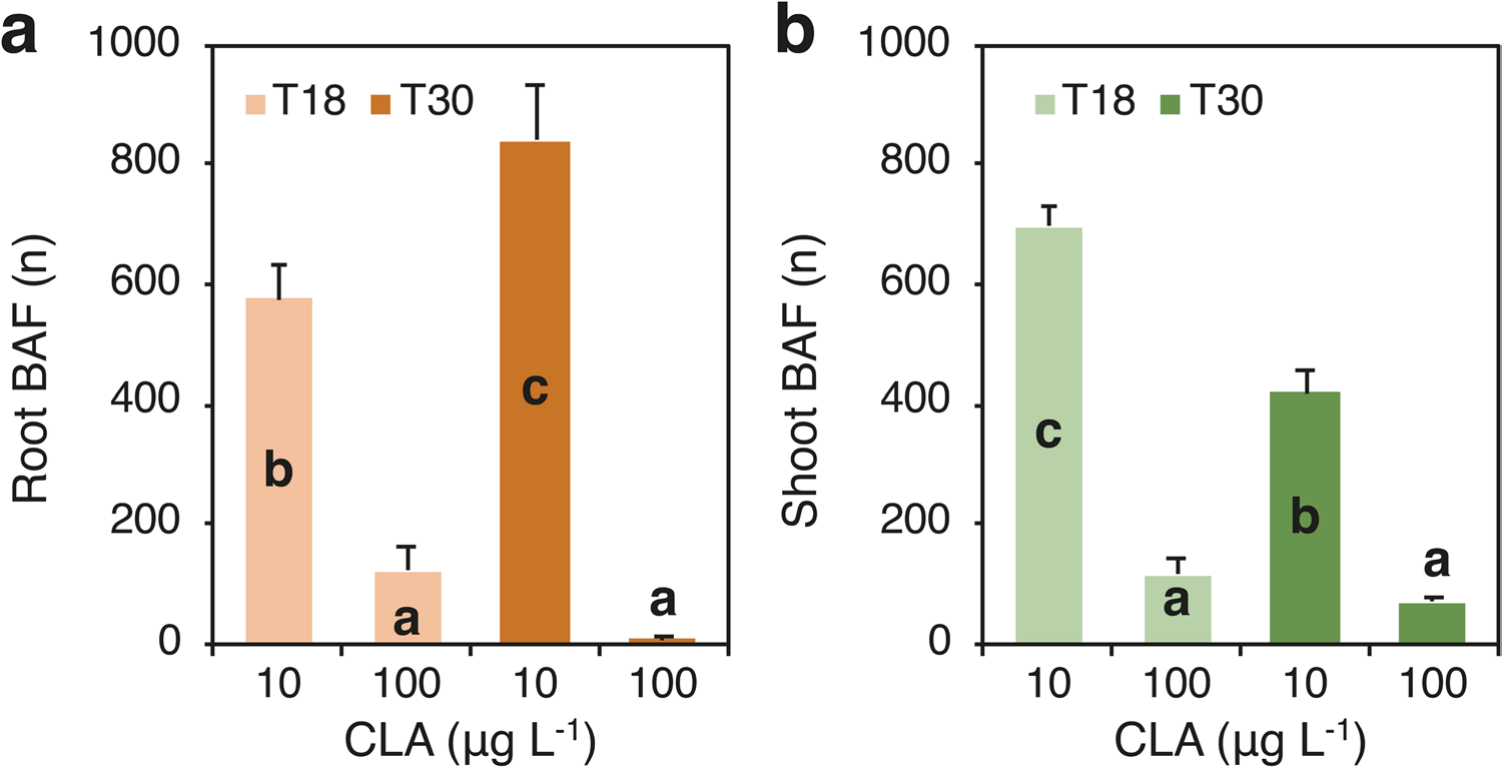
Effect of the interaction between clarithromycin (CLA) concentration in the nutrient medium (10 and 100 µg L.^−1^) (dose) and time of exposure (18 and 30 days: T18 and T30) on the bioaccumulation factor (BF) of CLA roots (**a**) and shoots (**b**) of *Arundo donax* L. Data are mean ± SE (*n* = 6). Different letters indicate significant differences at *P* ≤ 0.05. Details about the two-way ANOVAs are given in Supplementary Table S5

The bioaccumulation factor (BAF) of roots/shoots, calculated as the ratio of CLA concentration in roots/shoots (fresh weight basis) and in the nutrient solution, was significantly affected by the interaction between CLA dose and time of exposure (Fig. 5; Supplementary Table S5). Root BAF at both sampling times was significantly higher at 10 µg CLA L^−1^ compared to 100 µg CLA L^−1^ (ca. 708 *vs* 66), but the highest value was reached at T30 (ca. 814). Indeed, root BAF increased over time at the lower CLA dose, and decreased over time at the higher CLA dose, although differences in time of exposure at this dose were statistically not significant (Fig. 5a). Shoot BAF at both sampling times was higher at 10 µg CLA L^−1^ compared to 100 µg CLA L^−1^, decreased over time at the lower CLA dose, and it did not change with at the higher CLA dose (Fig. 5b). The translocation factor (TF) of CLA, calculated as the ratio of CLA concentration in shoots and roots was not affected by CLA dose and time of exposure (Supplementary Table S5). On average, TF of CLA was 1.0.

### Growth and uptake of DCF by Arundo donax

**Fig. 6:**
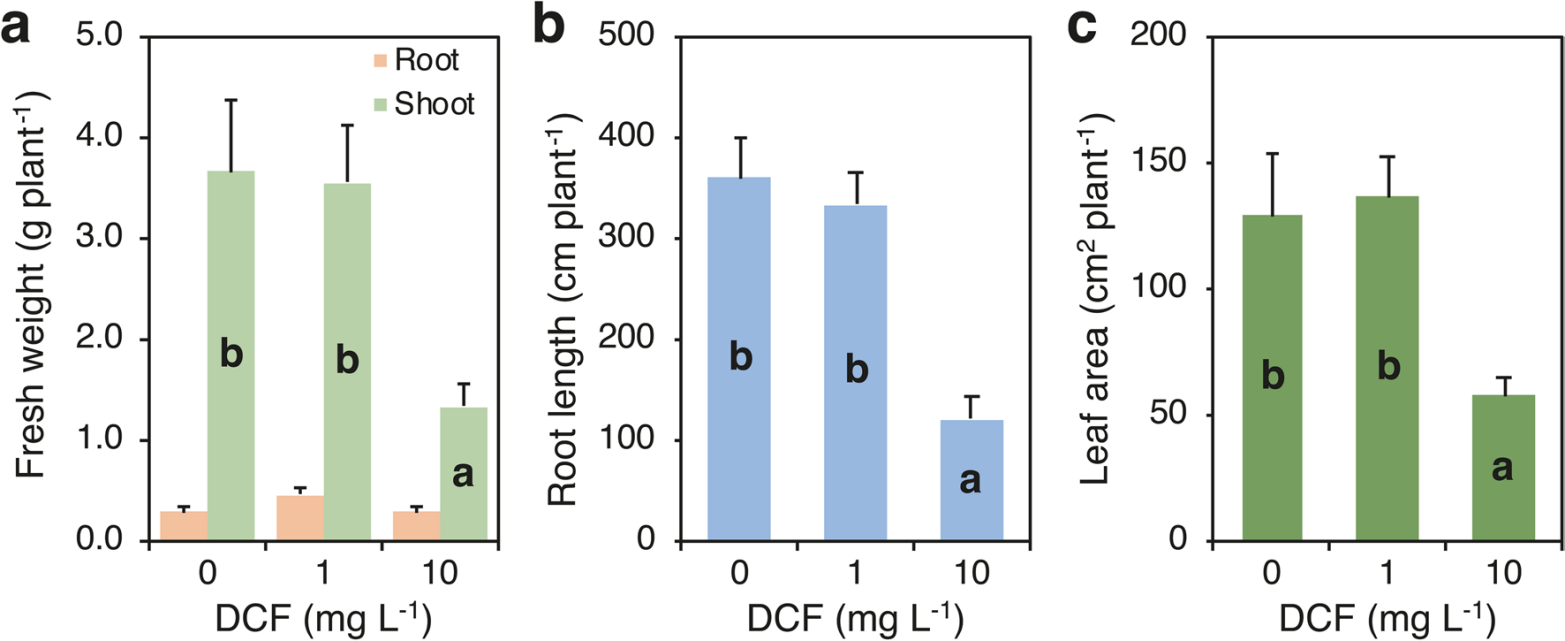
Effect of diclofenac (DCF) concentration in the nutrient medium (0, 1, and 10 mg L.^−1^) (dose) on fresh weight of shoots and roots (**a**), root length (**b**), and leaf area (**c**) of *Arundo donax*. Data are mean ± SE (*n* = 4). Different letters indicate significant differences at *P* ≤ 0.05. Details about the one-way ANOVAs are given in Supplementary Table S6

**Fig. 7:**
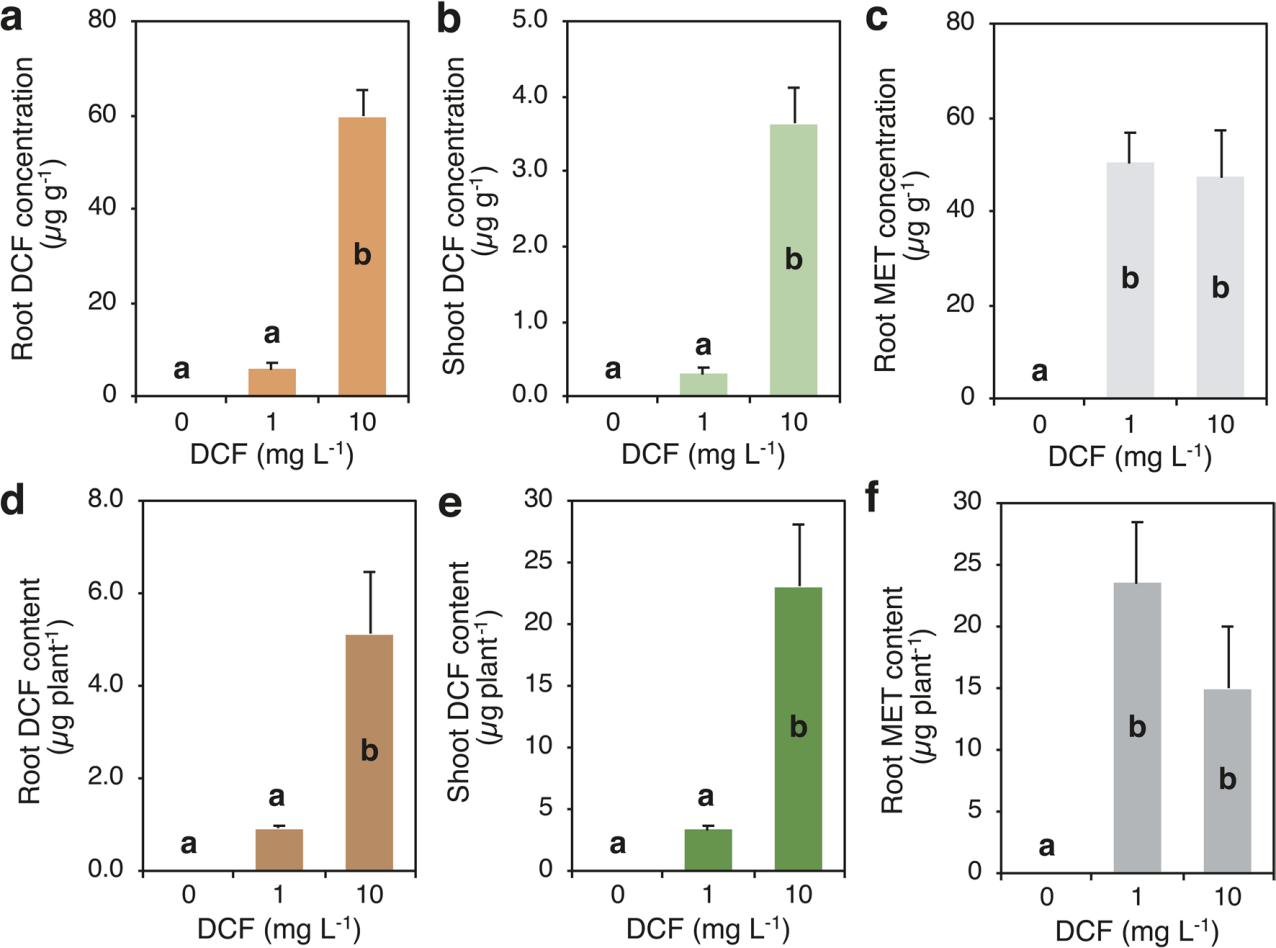
Effects of diclofenac (DCF) concentration in the nutrient medium (0, 1, and 10 mg L.^−1^) (dose) on DCF concentration and content in roots (**a**, **d**) and shoots (**b**, **e**) and on DCF metabolite (MET, 4′-hydroxydiclofenac) concentration and content in roots (**c**, **f**). Data are mean ± SE (*n* = 4). Different letters indicate significant differences at *P* ≤ 0.05. Details about the one-way ANOVAs are given in Supplementary Table S7

Similar to the experiment carried out to evaluate plant growth and CLA uptake, plants harvested just before the addition of DCF (T0) had an average of 1.2 culms per plants and 7.6 green leaves per plant, fresh weight was 1.05 g plant^−1^ (shoot plus roots) and plant height was 10 cm. After 18 days of exposure to DCF, shoot fresh weight, root length, and leaf area were affected by DCF and this effect varied according to its concentration in the medium (Fig. 6; Supplementary Table S6). Shoot fresh weight, root length, and leaf area at 1 mg DCF L^−1^ did not change compared to the untreated control, while they decreased at 10 mg DCF L^−1^ by 63%, 67%, and 55%, respectively. Conversely, root fresh weight, number of leaves, and stems and leaf SPAD readings were not affected by DCF exposure at both doses (Supplementary Table S6). The morphology of the root system of *A. donax* changed in response to DCF supply: in plants treated with 1 mg DCF L^−1^, the ratio root length/root fresh weight decreased by 80% compared with the untreated plants (from 1289 to 262 cm g^−1^), while in plants treated with 10 mg DCF L^−1^, the ratio was unchanged (230 cm g^−1^) (data not shown). Visual observations confirmed the effect of DCF at both doses on root architecture. DCF concentration and content in roots and shoots were affected by DCF concentration in the nutrient medium (Fig. 7; Supplementary Table S7). DCF was not detected in roots and shoots of the untreated plants, while concentration of DCF in roots and shoots increased with the increase of DCF dose (Fig. 7a, b). The increase from 1 to 10 mg DCF L^−1^ was about eightfold and over tenfold in roots and shoot, respectively. However, DCF concentration was much higher in roots than in shoot (about 16–19 times higher). Similarly, DCF content in roots and shoots increased due to the increase of DCF dose (4.6-fold in roots and 5.9-fold in shoot), but due to the higher biomass allocation to shoot compared with roots, DCF content in shoots was much higher than in roots (Fig. 7d, e). DCF content in the whole plant increased from 3.4 to 23 µg plant^−1^ with the increase of DCF dose from 1 to 10 mg L^−1^, which was partitioned for 79 and 82% into shoots, respectively, at the lower and higher DCF dose (Supplementary Table S7).metabolite concentration (MET: 4’-hydroxydiclofenac, 4’-OH DCF) was high in roots of plants treated with both DCF doses and was almost not detected in the shoots (Fig. 7c; Supplementary Table S7). However, no significant difference was detected between DCF doses (on average 48.9 µg g^−1^) (Fig. 7c) and values were similar to the DCF concentration observed in roots at the higher DCF dose (ca. 59.7 µg g^−1^) (Supplementary Table S7).

The bioaccumulation factor (BAF) of roots and shoots was not modified by DCF dose and was on average 59.3 and 3.1, respectively (Supplementary Table S8). Similarly, the translocation factor of DCF did not vary according to DCF dose and was on average 0.06, indicating that DCF concentration in shoots was ten times lower than in roots.

## Discussion

### Microbial degradation of CLA and DCF

In our batch experiments with the selected microorganisms, the experimental set-up allowed to exclude or minimize volatilization and photooxidation, and differences in removal efficiency can be exclusively attributed to the differential ability of the microorganisms to tolerate high CLA and DCF concentration and to use these compounds as C and energy sources or degrade them as a result of co-metabolism. In addition, the axenic growing conditions applied in our experiment prevent the occurrence of limitations to microbial growth due to competition with other microorganisms. Indeed, the growth of the ligninolytic basidiomycetes, such as *P. chrysosporium* and *T. versicolor*, in most soils is limited due to the low amount of available C and N, as some soil microbes are very efficient competitors at low resource availabilities (Baldrian 2008). Moreover, we tested higher concentrations of both CLA and DCF compared to the ones generally recorded in water bodies, since one of the key questions posed by the scientific community is whether PhCs could induce toxic effects to the microbial community putatively able to degrade contaminants and thus potentially able to reduce microbial degradation.

Overall, the highest removal efficiency of CLA, classified as a recalcitrant compound, was recorded with *P. chrysosporium*: the PhC was almost completely degraded (ca. 80%) already at 72 h. Conversely, the degradation ability of *T. versicolor* was lower, although lower rates were recorded at the higher CLA concentration. In comparison with these fungi, the bacterium *S. rochei* showed a lower degradation ability, not exceeding 50% of CLA at both doses. These results suggest that the different ability of the tested microorganisms to degrade CLA might be related to the capacity of producing significant amounts of extracellular laccases (Sutaoney et al. 2022). Accordingly, Margot et al. (2013) found that laccase activity produced by *T. versicolor* was more than 20 times greater than four strains of the bacterial genus *Streptomyces* (*S. cyaneus*, *S. ipomoea*, *S. griseus* and *S. psammoticus*).

The reduction of the degrading ability of *P. chrysosporium* at the highest CLA dose could be attributed to the toxicity of the compound to the fungus. Although in the present study we could not determine the degradation products of CLA, it is important to highlight that degradation could not result in a complete disappearance of toxicity, as some oxidative treatments of CLA yield a number of products, some of which (e.g., 14-hydroxy(R)-clarithromycin) have pharmacological activity, and thus, a putative toxicity for the microorganisms (Baumann et al. 2015). However, Zeng et al. (2021) studied the metabolic mechanism of CLA in waste activated sludge from anaerobic digestion system and demonstrated that CLA could be degraded into the macrolide antibiotic oleandomycin with lower antimicrobial activity and into other metabolites without antimicrobial activity. This indicates that effective degradation and reduced potential environment risk induced by CLA could be attained.

DCF is classified as a recalcitrant or poor removal compound following biological wastewater treatment processes (removal efficiency < 30%) (Matamoros and Bayona 2006; Luo et al. 2014). This behavior was attributed to the molecular properties of DCF, and specifically to the absence of electron donating groups in the molecule (Tran and Gin 2017). In our study, DCF removal was observed in all experiments, but the removal efficiency was highly variable (from 30 to 97%), according to the degrading microorganism and DCF concentration. Removal efficiency by *S. rochei* at the lower DCF concentration (ca. 30%) is similar to the values recorded by Tran and Gin (2017) and other authors (e.g., Zhang et al. 2008) in activated sludge processes, where microorganisms mineralize or transform pollutants. However, when DCF was applied at the higher concentration, the removal efficiency of *S. rochei* increased, but did not exceed 60%.

We can therefore assume that the high degrading ability of *S. rochei* at the higher CLA and DCF concentrations could result from bacterial metabolism using PhCs as C and energy sources. This hypothesis is supported by the high ability to remove DCF from liquid cultures by Actinobacteria endophytes (i.e., *Streptomyces*, *Microbacterium*, and *Glycomyces*) isolated from the roots and rhizomes of *Miscanthus* × *giganteus* plants (Sauvêtre et al. 2020). The highest DCF removal rates were observed for the isolates DS24 (41%) and DS4 (35%) both identified as *Streptomyces griseorubiginosus*, which were able to use DCF as a sole C source. Conversely, higher degrading ability of *T. versicolor* (Badia-Fabregat et al. 2015) and bacteria was recorded in activated sludge from municipal wastewater depuration plant with nutrient addition (Muter et al. 2017), supporting the general agreement that most of the pollutants are co-metabolically degraded (Harms et al. 2011).

Similar to the results obtained with CLA, degradation ability of DCF increased with *T. versicolor*, and even more with *P. chrysosporium* compared to *S. rochei*, although with the higher concentration of DCF in nutrient media both fungi required more time to achieve the same percentage of removal.

In many environmental conditions and applications, bacteria are chosen or self-established because they outperform fungi. Compared to fungi, bacteria tolerate a broader range of habitats, use higher specificity biochemical reactions, more often they productively degrade contaminants (leading to independence from auxiliary organic substrates), grow faster, and are more mobile in aqueous environments (Harms et al. 2011). Additionally, failure of filamentous fungi in remediation schemes (namely, land farming and soil reactors) have been reported, due to lack of supply of organic substrates, oxygen starvation, and mechanical disturbance sensitivity that prevents fungi from developing mycelia (Lamar et al. 1994). However, bacteria might be disadvantaged if substrates contain rare mineral elements, have a low bioavailability, contain little energy, or occur permanently at low concentrations. In addition, fungi possess other characteristics that could make their use more attractive than bacteria, such as long-range transport, production of many intracellular, and extracellular enzymes involved in chemical catabolism that lacks substrate specificity (Harms et al. 2011).

### Growth and uptake of CLA and DCF by Arundo donax

In CWs, plants mainly contribute to PhC removal through direct uptake, absorption, and sequestering of contaminants, promoting microbial growth around the roots, and controlling water movement (Ravichandran and Philip 2021). The use of non-conventional water resources, such as treated wastewater for irrigation purposes, is a consolidated practice worldwide, and the evidence of the occurrence of PhCs in waters has promoted research activity in order to elucidate the uptake and bioaccumulation of PhCs in the edible parts of food crops and fodders and their subsequent entry into the food chain. While food and feed crops were extensively tested for uptake and accumulation of contaminants (Christou et al. 2019), *A. donax* used as biomass plants, i.e., non-food crop plants grown for energy production, has not yet been tested for PhCs removal or biotransformation, despite the fact that energy crops pose no intake risks for humans and animals.

In our hydroponic experiments, we evaluated the ability of *A. donax* to uptake CLA and DCF from nutrient media and to PhCs partition the into shoots and roots. The experimental set-up allowed to exclude or minimize volatilization and photooxidation. The growth pattern of *A. donax* indicates that this species expresses a toxicity response to CLA only at the maximum dose of 100 µg L^−1^. Although shoot biomass production was not affected by CLA exposure at either dose, root growth and length were significantly affected (48% increase in weight and 27% decrease in length, compared to control, corresponding to more than 50% decrease of root length/weight ratio) with 100 µg CLA L^−1^. Therefore, this CLA dose dramatically changed root morphology, with short and thick main roots and the inhibition of fine lateral roots. To our knowledge, this is the first report of CLA producing a severe root length reduction and stunted roots formation.

CLA was found in roots and shoots at increasing concentrations with increasing CLA dose, but the values were similar in both roots and shoots for any dose (roots: 0.89–1.82 µg g^−1^; shoots: 0.77–1.21 µg g^−1^), and TF was consequently about 1. However, due to the different growth pattern of the plant parts, CLA content was much higher in shoots than in roots, suggesting that the plant is able not only to uptake CLA from the growth medium but also to translocate and accumulate it into the shoots. In previous studies, carried out in hydroponic culture, CLA was detected at concentrations up to 1.63 and 5.0 µg g^−1^ in lettuce leaves and roots, respectively (Tian et al. 2019). These higher values compared to our results could be determined by the small size and fast growth of lettuce, leading to the quick uptake and accumulation of CLA in plant tissues. However, in the study of Manasfi et al. (2021), the accumulation of CLA in lettuce leaves irrigated with spiked treated wastewater with 10 μg CLA L^−1^ was small (126.6 ng g^−1^ d.w., corresponding to 1.6 μg L^−1^ on fresh basis assuming 8% d.w. of leaves) and similar to our results. Furthermore, they were unable to detect any of the known metabolites of CLA, probably because they were present, but at values below the detection threshold. Conversely, in the work of Tian et al. (2019), eight metabolites of CLA were detected in both lettuce leaves and roots after 18 days of exposure, and their proportion to the parent compound was estimated to be greater than 70%, indicating that most of the CLA was metabolized in plant tissues. Although in the present research we did not determine degradation products of CLA in tissues of *A. donax*, we cannot exclude their presence.

DCF treatments affected plant growth only at the highest dose, with which shoot fresh weight and root length were severely reduced. In contrast to the results with CLA treatments, shoots showed reduced growth and toxic effect, although morphological modification of the root system with many short and stunted roots occurred also with DCF exposure at both doses. DCF was found in roots and shoots at increasing concentrations with increasing DCF dose, but the concentrations were much higher in roots than in shoots (16–19 times higher) and the values were similar in roots and shoots for each rate (roots: 6–60 µg g^−1^, shoots: 0.3–3.6 µg g^−1^), and TF was consequently about 0.1. A higher DCF concentration in roots than in shoots agrees with the results of Ravichandran and Philip (2021) in *Cannabis indica* and *Cannabis zizanioides* and of Zhang et al. (2013) in *Scirpus validus*. In the present study, the 4′-OH DCF metabolite was detected in roots and not in shoots and its proportion to the parent compound varied according to the DCF dose. We estimated that 4′-OH DCF was approximately 88% and 39% of the sum of DCF and 4′-OH DCF metabolite content, at 1 and 10 mg DCF L^−1^, respectively. These results suggest that most of the DCF was metabolized in root tissues, and as expected, the rate of degradation was higher at the lower dose. Similar accumulation of the metabolites 4′-OH DCF and DCF-OH glucose was detected in roots by Ravichandran and Philip (2021).

Previous studies have shown reduced plant growth due to DCF exposure (e.g., Ziółkowska et al. 2014; Schmidt and Redshaw 2015; Podio et al. 2020). Shortening of root and shoot lengths was detected on three legume plants (pea, lupin, and lentil) when the DCF concentration increased from 17.8 to 3560 mg L^−1^ and the negative effect increased with the increase of the DCF dose (Ziółkowska et al. 2014). Schmidt and Redshaw (2015) found a negative effect of DCF at 1 mg L^−1^ on the ratio root to aerial part biomass of *Raphanus sativus*. Conversely, Pierattini et al. (2018) observed no differences in leaves number, shoot length, and fresh or dry weight of poplar plants (*Populus alba*, L. Villafranca clone) exposed to 0 and 1 mg L^−1^ of DCF in hydroponic solution. Similarly, Podio et al. (2020) did not found differences in phenotypes of chicory plants exposed to 0 and 1 mg DCF L^−1^ during seed germination and early growth stages, but found a negative effect of DCF on the concentration of photosynthetic pigments and an activation of the plant detoxification system. Other authors reported induction of other enzymes following DCF exposure (i.e., catalase, glycosyltransferase and glutathione S-transferase) (Bartha et al. 2014; Huber et al. 2016; Majewska et al. 2018; Pierattini et al. 2018). Overall, these studies showed that the effect of DCF on plants depends on DCF dose and sensitivity can change according to plant species, and suggested that high doses can cause oxidative stress, mainly in roots, generating phenotypic changes and activating endogenous antioxidant defense mechanisms.

Plant uptake is thought to be strongly dependent on the physicochemical characteristics of the compound, including Henry’s Law constant, water solubility, and octanol–water partition coefficient (hydrophobicity; Kow). Dissociation constants are important because they can describe whether a compound is neutral or ionizable at environmentally relevant pH values. Previous research demonstrated that plant uptake of dissociated species of an ionizable compound is lower compared to a neutral compound (Malchi et al. 2014). There are separate models for predicting chemicals uptake in both of these forms (Trapp 2000; Trapp et al. 2010). This is because the uptake of a neutral compound can be mainly related to octanol–water partition coefficient, whereas the uptake of an ionic compound depends on pH in the external solution and the transport depends also on ion trapping in the phloem and electrostatic interactions with cell walls. In this study, CLA and DCF are expected to be ionizable in the hydroponic solution (pKa equal to 8.99 and 4.15, respectively). Therefore, these PhCs are expected to exist in the cation and anion form, respectively, in the environment at pH values from 5 to 9 (Pubchem 2022). Organic chemicals with log Kow > 4 are expected to have high potential for root retention and low translocation capacity. The lower log Kow of CLA (3.16) compared to the log Kow of DCF (4.5) could explain why CLA is more easily translocated and accumulated mainly in leaves following the water transpiration current (Duarte-Davidson and Jones 1996). However, these results disagree with the general belief that cationic compounds (in our study CLA) had significantly higher accumulation in roots and significantly lower accumulation in leaves than anionic compounds (DCF) (Wu et al. 2013; Dodgen et al. 2015; Miller et al. 2016).

The low translocation factor is a favorable character for edible plants, except for crops yielding roots (i.e., sugarbeet, carrot, radish), since PhCs are mainly concentrated in roots that are not harvested and remain in soil as residues. Conversely, the low translocation factor is an unfavorable character for energy crops whose aerial plant part is harvested for energy production and the PhCs eventually accumulated in shoots do not interfere with the transformation process into gaseous/liquid fuels and are easily degraded during these processes. In perennial crops, the accumulation of PhCs into rhizomes at concentrations above the toxicity thresholds could hinder regrowth in the following year.

## Conclusions

This study demonstrates that *S. rochei*, *T. versicolor*, and especially *P. chrysosporium* are able to break down CLA and DCF in liquid media to complete elimination, suggesting that these microorganisms are suitable candidates for application in the remediation of CLA and DCF-contaminated waters. Furthermore, according to the obtained results, *A. dona*x was shown to behave as accumulator for CLA and DCF. In accordance with the physicochemical characteristics of the PhCs, CLA was mainly translocated from roots and concentrated in leaves, while DCF was mainly accumulated in roots. Therefore, the association of *A. dona*x with microbial inoculants promises to improve the efficiency of bioremediation systems. However, to improve the design of cost-effective degradation systems for their better performance, we would need to better clarify which transformation products are formed, which are the microbial- and plant-mediated biotransformation pathways, and if the microbial processes are driven by co-metabolism and/or catabolism.

## Supplementary Information

Below is the link to the electronic supplementary material.

Supplementary file1 (DOCX 42 KB)

## Data Availability

Supplementary material is available. Raw data will be made available on request.
