# Supplementary material for: Effective bioremediation of clarithromycin and diclofenac in wastewater by microbes and Arundo donax L
Source: Environ Sci Pollut Res Int. 2023 May 30;30(31):77193–209. doi: 10.1007/s11356-023-27660-4 (PMC10300175; doi:10.1007/s11356-023-27660-4)
Supplement: Supplementary file 1 — Supplementary file1 (DOCX 42 KB) [file 11356_2023_27660_MOESM1_ESM.docx]

**Effective bioremediation of clarithromycin and diclofenac in wastewater by microbes and *Arundo* donax L.**

Laura Ercoli^1^, Rudy Rossetto^1^, Sabrina Di Giorgi^2^, Andrea Raffaelli^1^, Marco Nuti^1^, Elisa Pellegrino^1,*^

^1^Crop Science Research Center (CSRC), Scuola Superiore Sant'Anna, Piazza Martiri della Libertà 33, 56127 Pisa, Italy.

^2^Ministero della Salute, Direzione Generale per l’Igiene e la Sicurezza degli Alimenti e della Nutrizione, Roma, Italy.

^*^Correspondence: [elisa.pellegrino@santannapisa.it](mailto:elisa.pellegrino@santannapisa.it); Phone: 0039 050 883181.

| **Table S1** Selection Reaction monitoring (SRM) transitions and retention times of the studied analytes. | | | | | | | |
| --- | --- | --- | --- | --- | --- | --- | --- |
| SRM Transition^a^ | Prec. Ion^b^ | Prod. Ion | DP (V) | FP (V) | CE (eV) | CXP (V) | RT (min) |
| DCF Qual^c^ | 296 | 250.2 | 0 | 117 | 16 | 4.6 | 7.05 |
| DCF Quant^d^ | 296 | 214.2 | 0 | 117 | 46.5 | 5.9 | 7.05 |
| 4-OH-DCF Quant^d^ | 312.1 | 230 | 3 | 146 | 44 | 1.8 | 6.32 |
| 4-OH-DCF Qual^c^ | 312.1 | 265.9 | 3 | 146 | 19 | 6 | 6.32 |
| CLA Quant^d^ | 748.6 | 158.2 | 16 | 180 | 40 | 3.8 | 5.73 |
| CLA Qual^c^ | 748.6 | 590.5 | 16 | 180 | 25 | 10.8 | 5.73 |
| ^a^ Source parameters: Nebulizer gas (NEB, air), 12 (arbitrary units); curtain gas (CUR, nitrogen), 7 (arbitrary units); temperature (TEM), 400°C; ionspray voltage (IS), 5500 V; collision gas (CAD, nitrogen), 6 (arbitrary units). | | | | | | | |
| ^b^ Prec. Ion: recursor Ion; Prod. ion: Product Ion; DP: Declustering Potential; FP: Focusing Potential; CE: Collision Energy; CXP: Collision cell eXit Potential; RT: Retention Time, Selected Reaction Monitoring; | | | | | | | |
| ^c^ SRM transition used for qualitative confirmation of the analyte identity; | | | | | | | |
| ^d^ SRM transition used for quantitation. | | | | | | | |

| **Table S2** *P*-values of two-way ANOVAs evaluating the effect of PhC concentration (Dose) and incubation time (T_inc_) on the removal rate (%) of diclofenac (DCF) and clarithromycin (CLA) in the nutrient medium. | | | | | | | | |
| --- | --- | --- | --- | --- | --- | --- | --- | --- |
| Microorganism/factor | *Streptomyces rochei* | |  | *Phanaerochete chrysosporium* | |  | *Trametes versicolor* | |
|  | CLA | DCF |  | CLA | DCF |  | CLA | DCF |
| Dose^a^ | **0.001^c^** | **< 0.001** |  | **0.045** | **0.034** |  | 0.181 | **<0.001** |
| T_inc_^b^ | **<0.001** | **<0.001** |  | **<0.001** | **<0.001** |  | **<0.001** | **<0.001** |
| Dose x T_inc_ | 0.145 | **0.018** |  | 0.324 | **<0.001** |  | **0.006** | **0.002** |
| ^a^ CLA: 10 and 100 µg L^-1^; DCF: 1 and 10 mg L^-1^; | | | | | | | | |
| ^b^ 0, 20, 48, 72, 144 h; | | | | | | | | |
| ^c^ In bold statistically significant values (*P* ≤ 0.05). At least three replicates per treatment. | | | | | | | | |

| **Table S3** Total plant fresh weight (FW), root FW, shoot FW, root length, leaf area, leaf and stem number and chlorophyll meter (SPAD) data of *Arundo donax* L. treated with 0 (control), 10 µg L^-1^ and 100 µg L^-1^ of clarithromycin (CLA) and sampled at 18 and 30 days of growth after the beginning of the experiment (T18 and T30, respectively). *P*-values of the two-way ANOVA testing the effect of CLA dose and time are also reported. | | | | | | | | | |
| --- | --- | --- | --- | --- | --- | --- | --- | --- | --- |
| CLA dose (µg L^-1^) | Time | Total FW (g) | Root FW (g) | Shoot FW (g) | Root length (cm) | Leaf area (cm^2^) | Leaves (n) | Stems (n) | SPAD (unit) |
| 0 | 18 d | 3.01 ± 0.43^a^ | 0.35 ± 0.11 | 2.66 ± 0.37 | 529.0 ± 50.3 | 122.7 ± 21.8 | 11.90 ± 0.81 | 1.25 ± 0.16 | 40.5 ± 0.5 a |
| 10 | 18 d | 4.71 ± 1.34 | 0.46 ± 0.11 | 4.25 ± 1.24 | 696.8 ± 85.2 | 150.5 ± 38.3 | 14.68 ± 1.02 | 1.50 ± 0.09 | 42.5 ± 0.8 ab |
| 100 | 18 d | 3.67 ± 0.19 | 0.62 ± 0.09 | 3.05 ± 0.13 | 373.3 ± 60.2 | 117.7 ± 6.9 | 12.83 ± 0.83 | 1.33 ± 0.14 | 42.6 ± 0.6 ab |
| 0 | 30 d | 5.89 ± 0.48 | 1.33 ± 0.17 | 4.56 ± 0.31 | 793.2 ± 26.1 | 155.5 ± 11.4 | 17.33 ± 1.81 | 1.83 ± 0.40 | 44.8 ± 1.1 b |
| 10 | 30 d | 5.78 ± 0.36 | 1.34 ± 0.07 | 4.44 ± 0.33 | 787.6 ± 15.2 | 151.7 ± 12.8 | 16.25 ± 2.48 | 1.25 ± 0.16 | 44.1 ± 1.2 b |
| 100 | 30 d | 5.59 ± 0.48 | 1.86 ± 0.17 | 3.73 ± 0.32 | 597.2 ± 50.0 | 136.5 ± 10.3 | 13.84 ± 0.55 | 1.42 ± 0.16 | 42.2 ± 0.4 ab |
|  |  |  |  |  |  |  |  |  |  |
| *P*-value CLA dose |  | 0.463^b^ | **0.010** | 0.246 | **0.001** | 0.497 | 0.341 | 0.663 | 0.557 |
| *P*-value Time |  | **0.002** | **<0.001** | 0.064 | **<0.001** | 0.294 | **0.033** | 0.426 | **0.015** |
| *P*-value CLA dose x Time |  | 0.399 | 0.327 | 0.329 | 0.300 | 0.732 | 0.264 | 0.164 | **0.036** |
| ^a^ Values are means ± SE of six replicates; | | | | | | | | | |
| ^b^ *P*-values in bold indicate a statistical significant difference according to two-way ANOVA, testing the treatments CLA dose and time, used as fixed factors, and following the post-hoc Tukey-B test (*P*≤0.05). For SPAD, numbers followed by different letters are significantly different, according to the significance of the interaction between CLA dose and time. Results of the significant main effects of CLA dose and time and of interaction dose x time are reported in Fig. 3. In Fig. 3, for shoot FW, the main effects of CLA dose and time are also reported. | | | | | | | | | |

| **Table S4** Root, shoot and total clarithromycin (CLA) concentration and content of *Arundo donax* L. treated with 0 (control), 10 µg L^-1^ and 100 µg L^-1^ of CLA at 18 and 30 days of growth after the beginning of the experiment (T18 and T30, respectively). Concentration data are expressed for fresh dry weight. *P*-values of the two-way ANOVA testing the effect of CLA dose and time are also reported. | | | | | | |
| --- | --- | --- | --- | --- | --- | --- |
| CLA dose (µg L^-1^) | Time | Root CLA conc. (µg g^-1^) | Shoot CLA conc. (µg g^-1^) | Root CLA content (µg plant^-1^) | Shoot CLA content (µg plant^-1^) | Total CLA content (µg plant^-1^) |
| 0 | 18 d | 0.00 ± 0.00^a^ a | 0.00 ± 0.00 | 0.00 ± 0.00 a | 0.00 ± 0.00 | 0.00 ± 0.00 |
| 10 | 18 d | 1.16 ± 0.09 c | 1.06 ± 0.05 | 0.53 ± 0.16 b | 4.51 ± 2.18 | 5.04 ± 1.16 |
| 100 | 18 d | 1.82 ± 0.08 d | 1.80 ± 0.43 | 1.13 ± 0.21 c | 5.49 ± 2.81 | 6.62 ± 1.66 |
| 0 | 30 d | 0.00 ± 0.00 a | 0.00 ± 0.00 | 0.00 ± 0.00 a | 0.00 ± 0.00 | 0.00 ± 0.00 |
| 10 | 30 d | 0.89 ± 0.05 b | 0.77 ± 0.04 | 1.19 ± 0.07 c | 3.42 ± 1.70 | 4.61 ± 0.26 |
| 100 | 30 d | 0.98 ± 0.08 bc | 1.21 ± 0.10 | 1.82 ± 0.13 d | 4.51 ± 2.22 | 6.34 ± 0.49 |
|  |  |  |  |  |  |  |
| *P*-value CLA dose |  | **<0.001**^b^ | **<0.001** | **<0.001** | **<0.001** | **<0.001** |
| *P*-value Time |  | **<0.001** | 0.063 | **<0.001** | 0.267 | 0.705 |
| *P*-value CLA dose x Time |  | **<0.001** | 0.289 | **0.016** | 0.718 | 0.956 |
| ^a^ Values are means ± SE of six replicates; | | | | | |  |
| ^b^ *P-*values in bold indicate a statistical significant difference according to two-way ANOVA testing the treatments CLA dose and time, used fixed factors and following the post-hoc Tukey-B test (*P≤*0.05). For root CLA concentration and content, numbers followed by different letters are significantly different, according to the significance of the interaction between CLA dose and time. Results of the significant main effect CLA dose and of the interaction dose x time are reported in Fig. 4. | | | | | | |

| **Table S5** Bioaccumulation factor (BF) in roots and shoot and translocation factor (TF) of clarithromycin (CLA) of *Arundo donax* L. treated with 10 µg L^-1^ and 100 µg L^-1^ of CLA at 18 and 30 days of growth after the beginning of the experiment (T18 and T30, respectively). *P*-values of the two-way ANOVA testing the effect of CLA dose and time are also reported. | | | | |
| --- | --- | --- | --- | --- |
| CLA dose (µg L^-1^) | Time | Root BAF (n) | Shoot BAF (n) | TF (n) |
| 10 | 18 d | 574.3 ± 61.6^a^ b | 698.1 ± 33.3 c | 0.93^a^ ± 0.07 |
| 100 | 18 d | 121.6 ± 40.7 a | 114.8 ± 28.9 a | 0.97 ± 0.20 |
| 10 | 30 d | 841.2 ± 90.4 c | 418.3 ± 36.0 b | 0.88 ± 0.09 |
| 100 | 30 d | 9.8 ± 0.8 a | 67.4 ± 9.4 a | 1.23 ± 0.01 |
|  |  |  |  |  |
| *P*-value CLA dose |  | **< 0.001**^b^ | **< 0.001** | 0.110 |
| *P*-value Time |  | 0.209 | **< 0.001** | 0.361 |
| *P*-value CLA dose x Time |  | **0.007** | **0.002** | 0.203 |
| ^a^ Values are means ± SE of six replicates; | | | | |
| ^b^ *P*-values in bold indicate a statistical significant difference according to two-way ANOVA testing the treatments CLA dose and time, used fixed factors and following the post-hoc Tukey-B test (*P*≤0.05). For root and shoot BAF, numbers followed by different letters are significantly different, according to the significance of the interaction between CLA dose and time. Results of the significant interaction CLA dose x time are reported in Fig. 5. | | | | |

| **Table S6** Total plant fresh weight (FW), root FW, shoot FW, root length, leaf area, leaf and stem number, chlorophyll meter (SPAD) data of *Arundo donax* L. treated with 0 (control), 1 and 10 µg L^-1^ of diclofenac (DCF) at 18 days of growth after the beginning of the experiment. *P*-values of the the one-way ANOVA testing the effect of DCF dose are also reported. | | | | | | | | |
| --- | --- | --- | --- | --- | --- | --- | --- | --- |
| DCF dose (mg L^-1^) | Total FW (g) | Root FW (g) | Shoot FW (g) | Root length (cm) | Leaf area (cm^2^) | Leaves (n) | Stems (n) | SPAD (unit) |
| 0 | 3.96^a^ ± 0.76^a^ b | 0.28 ± 0.05 | 3.68 ± 0.71 b | 360.8 ± 41.6 b | 129.6 ± 24.4 b | 13.0 ± 1.6 | 2.5 ± 0.4 | 28.3 ± 0.9 |
| 1 | 4.02 ± 0.63 b | 0.46 ± 0.07 | 3.56 ± 0.57 b | 334.9 ± 32.2 b | 136.9 ± 15.6 b | 12.3 ± 0.6 | 2.0 ± 0.1 | 26.8 ± 0.8 |
| 10 | 1.62 ± 0.26 a | 0.29 ± 0.04 | 1.36 ± 0.23 a | 120.6 ± 22.4 a | 58.0 ± 7.4 a | 10.4 ± 1.1 | 2.4 ± 0.3 | 26.8 ± 1.3 |
| *P*-value^b^ | **0.017**^b^ | 0.054 | **0.013** | **< 0.001** | **0.010** | 0.310 | 0.503 | 0.488 |
| ^a^ Values are means ± SE of four replicates; | | | | | | | | |
| ^b^ *P*-values in bold indicate a statistical significant difference according to one-way ANOVA, testing the DCF dose as fixed factor, and following the post-hoc Tukey-B test (*P*≤0.05). For total, shoot and root FW and leaf area, numbers followed by different letters are significantly different. Results of the significant effect of DCF dose are reported in Fig. 6. | | | | | | | | |

| **Table S7** Roots, shoots, and total diclofenac (DCF) and DCF metabolite (MET: 4’-hydroxy DCF, 4’-OH DCF) concentrations and content of *Arundo donax* L. treated with 0 (control), 1 mg L^-1^ and 10 mg L^-1^ of DCF at 18 days of growth after the beginning of the experiment. Concentration data are expressed for fresh dry weight. *P*-values of the one-way ANOVA testing the effect of DCF dose are also reported. | | | | | |
| --- | --- | --- | --- | --- | --- |
| DCF concentration  (mg L^-1^) | Root conc. (µg g^-1^) | Shoot conc. (µg g^-1^) | Root content (µg plant^-1^) | Shoot content (µg plant^-1^) | Total content (µg plant^-1^) |
|  | DCF | | | | |
| 0 mg L^-1^ | 0.00 ± 0.00^a^ a | 0.00 ± 0.00 a | 0.00 ± 0.00 a | 0.00 ± 0.00 a | 0.00 ± 0.00 a |
| 1 mg L^-1^ | 5.98 ± 1.09 a | 0.31 ± 0.08 a | 2.75 ± 0.20 a | 1.10 ± 0.06 a | 3.85 ± 0.23 a |
| 10 mg L^-1^ | 59.73 ± 5.47 b | 3.64 ± 0.47 b | 17.32 ± 3.82 b | 4.95 ± 1.36 b | 22.07 ± 5.13 b |
| *P*-value^b^ | **< 0.001**^b^ | **< 0.001** | **< 0.001** | **0.001** | **< 0.001** |
|  | MET | | | | |
| 0 mg L^-1^ | 0.00 ± 0.00 a | 0.00 ± 0.00 | 0.00 ± 0.00 a | 0.00 ± 0.00 | 0.00 ± 0.00 a |
| 1 mg L^-1^ | 50.44 ± 6.46 b | 0.00 ± 0.00 | 23.20 ± 4.96 b | 0.00 ± 0.00 | 23.20 ± 4.96 b |
| 10 mg L^-1^ | 47.27 ± 10.03 b | 0.03 ± 0.01 | 13.71 ± 5.03 b | 0.04 ± 0.01 | 13.75 ± 5.01 b |
| *P*-value | **< 0.001** | 0.391 | **0.003** | 0.391 | **0.003** |
| ^a^ Values are means ± SE of four replicates; | | | | | |
| ^b^ *P*-values in bold indicate a statistical significant difference according to one-way ANOVA, testing the DCF dose as fixed factor, and following the post-hoc Tukey-B test (*P*≤0.05). For all parameters with the exception of MET shoot conc. and content, numbers followed by different letter are significantly different. Results of the significant effect of DCF dose are reported in Fig. 7. | | | | | |

| **Table S8** Bioaccumulation factor (BF) in roots and shoot and translocation factor (TF) of diclofenac (DCF) of *Arundo donax* L. treated with 1 mg L^-1^ and 10 mg L^-1^ of DCF at 18 days of growth after the beginning of the experiment. *P*-values of the one-way ANOVA testing the effect of DCF dose are also reported. | | | |
| --- | --- | --- | --- |
| DCF concentration  (mg L^-1^) | Root BAF (n) | Shoot BAF  (n) | TF  (n) |
| 1 | 65.33 ± 11.41^a^ | 2.94 ± 0.79 | 0.05^a^ ± 0.00 |
| 10 | 53.32 ± 9.80 | 3.17 ± 0.70 | 0.06 ± 0.00 |
|  |  |  |  |
| *P*-value | 0.443^b^ | 0.838 | 0.069 |
| ^a^ Values are means ± SE of four replicates; | | | |
| ^b^ *P*>0.05 according to one-way ANOVA and the post-hoc Tuckey-B test. | | | |
